# Impact of an HIV Prevention Intervention on HIV Risk Behavior and Sexually Transmitted Infection among Female Sex Workers in Tamil Nadu, India

**DOI:** 10.4236/wja.2017.73014

**Published:** 2017-07-28

**Authors:** Joseph D. Williams, Arumugam Vijayaraman, Priya Krishnaswamy, Niranjan Saggurti, Sowmya Ramesh, Deepika Ganju

**Affiliations:** 1 Voluntary Health Services, Chennai, India; 2 Population Council, New Delhi, India

**Keywords:** HIV, Intervention Exposure, Female Sex Workers, STI, Tamilnadu, Condom Use

## Abstract

**Background:**

In 2003 a large-scale HIV prevention program was launched for key populations in six high HIV prevalence states of India. This paper assesses the effect of exposure to the intervention on condom use with commercial clients and experiences of sexually transmitted infection (STI) among female sex workers (FSWs) in Tamilnadu, a southern Indian state, between 2006 and 2009.

**Methods:**

Data were drawn from two rounds of cross-sectional surveys conducted in 2006 (N = 2010) and 2009 (N = 2500) among FSWs in Tamilnadu, recruited through probability-based sampling. A series of multivariate logistic regression models were constructed to examine the association between exposure to the intervention and change over time with condom use and self-reported STI. All the analyses were performed using STATA 11.1.

**Results:**

Overall, 48% of FSWs in 2006 and 90% in 2009 reported exposure to the intervention. Compared to 2006, there was a considerable increase in the proportion of FSWs reporting consistent condom use with regular and occasional clients at follow-up (2009). Further, the increase in consistent condom use over time with occasional (adjusted OR = 3.53, 95% CI: 2.47 - 5.05) and regular clients (adjusted OR = 4.97, 95% CI: 3.43 - 7.16) was significantly higher among FSWs exposed to the intervention than those not exposed. Additionally, a significant decline was observed in self-reported STI overtime among FSWs exposed to the intervention compared to their counterparts (adjusted OR = 0.39, 95% CI: 0.26 - 0.59).

**Conclusion:**

The HIV prevention program in Tamilnadu resulted in increased consistent condom use and a decrease in self-reported STI among FSWs exposed to intervention. These findings suggest that HIV prevention programs should aim to saturate coverage among key populations to sustain the gains achieved.

## Introduction

National adult (15 - 49 years) HIV prevalence in India is estimated at 0.26% (2015) [1]. The HIV epidemic in the country is heterogeneous, with some regions and populations showing disproportionately higher incidence [2]. Most HIV infections occur through heterosexual transmission, with unprotected paid sex being the major route of transmission particularly in the high HIV prevalence states of southern India [2]. Reducing the transmission of HIV and other sexually transmitted infections (STI) remains a public health priority.

In India the HIV epidemic is concentrated among key populations—female sex workers (FSWs), high-risk men who have sex with men/transgender persons (MSM/TG) and injecting drug users (IDUs), and since 1992 the National AIDS Control Organization (NACO) has been implementing targeted interventions for high risk groups and bridge populations to contain the epidemic in the high HIV prevalence states [3]. Alongside these efforts, in 2003, Avahan was launched in six high HIV prevalence states in India for high-risk groups to provide scaled up HIV prevention services through state-level providers and local non-governmental organizations (NGOs) [4].

In 2003, the Tamilnadu AIDS Initiative (TAI) was initiated under Avahan, through the NGO Voluntary Health Services (VHS), to scale up HIV prevention services in selected districts and saturate coverage among FSWs and MSM/TGs in Tamilnadu, a high HIV prevalence state in southern India [5]. Intervention districts in the state were selected in consultation with the Indian government to complement their prevention efforts [6] [7]. TAI-VHS’s objectives were to reduce STI in the target population, promote safer sex behaviors and address the structural barriers underlying HIV vulnerability. To date, there is little published evidence on the effect of the TAI-VHS intervention on HIV risk among key populations in the state of Tamilnadu. This paper describes the TAI-VHS HIV prevention intervention, and examines the effect of intervention exposure between baseline (2006) and follow-up (2009) on condom use behavior and self-reported STI among FSWs in Tamilnadu, India.

### The TAI-VHS HIV Prevention Program

Effective HIV prevention requires strategies that reduce the HIV vulnerability of high-risk groups [8]. The TAI-VHS HIV prevention program in Tamilnadu provided a proven package of services to address both the proximate and distal determinants of HIV risk. Services included peer education and outreach; condom distribution and social marketing; STI treatment; and structural interventions and community mobilization to address the distal determinants of HIV risk, such as violence and stigma [4] [9].

The program was community-led, with the community taking the lead in planning, designing, implementing and monitoring program activities [10] [11]. The program was delivered through community committee members, community advisors and peer outreach workers. Community committee members were elected representatives who resolved community issues, worked with NGOs to improve service uptake, helped to form community collectives, monitored program activities, and reviewed program implementation at review meetings. Community advisors, who were community leaders, helped to design program activities, reviewed the uptake of program services, make field visits, highlighted issues to be resolved and ensured service delivery [9].

Peer-led outreach programs are based on the principle that peers can strongly influence an individual’s behavior [12]. Peer educators, locally known as Peer Jeevans, were selected based on their interest and willingness to engage in outreach activities and were trained in communication and counselling skills. The ratio of peer educators to key populations was 1:30. Peer educators prepared micro-plans with inputs from outreach workers to prioritize delivery of services, and supported their peers to access program services. In addition to providing counselling on risk reduction and the importance of regular medical check-ups, they addressed misconceptions regarding condom use, promoted STI screening and early treatment, and provided referral services for HIV/STI testing and STI care to project-run clinics. Consistent condom use was promoted through condom “demo, re-demo” exercises, and using role play and counselling, condom negotiation skills were developed. Condoms were distributed weekly based on a quarterly condom gap analysis (by calculating the average number of sex acts per day and number of working days in a month, based on which condoms were distributed). Additionally, condoms were provided at program-run clinics. HIV-positive FSWs were encouraged to regularly visit antiretroviral therapy (ART) centers and adhere to treatment. Peer educators also addressed the needs and concerns of FSWs who experienced violence through crisis counselling, resolved disputes on inheritance and marital issues, and facilitated access to social entitlements and financial services.

The program aimed to increase the accessibility of STI services, provide quality care and early STI diagnosis and treatment to reduce infection among FSWs. As community members were hesitant to access STI care from government clinics due to stigma and discrimination, project-run clinics, including full-time clinics and satellite clinics, were set up in places that community members could easily access. Drop-in centers (DIC) were established in each clinic to provide a safe space for the community as well as counselling and condom supplies. By 2006, 41 program-owned STI clinics, five extended clinics and 12 referral clinics were operating in the state. Clinics regularly screened FSWs for STI, provided syndromic case management and referrals to government hospitals where necessary. FSWs’ partners and children were also provided treatment for general ailments. Clinic doctors and staff were sensitized to the needs of key populations and to provide care with empathy, and in each clinic, a committee comprising three community members was set up to provide information on available services and to address community fears regarding internal examinations and drawing of blood for testing. Community members assisted in daily medical and administrative activities at project clinics and DICs. Peer educators were trained and mentored by the clinic nurse, and served as clinic assistants when required. A community liaison officer was appointed at each clinic to ensure clients returned for follow-up visits. At government-run clinics, separate “safe” spaces for communities called “health hubs” were set up. Committee members who staffed the health hubs supported their peers to access clinic services; at the same time, videos and IPC materials were screened/displayed at the health hubs to build acceptance of the community among health staff. Condoms were also distributed at the health hubs.

To address violence, which was widespread among FSWs, a community-led crisis management wing was set up, staffed by informed and empowered key populations, popularly called Araichimani (meaning “Bell of Distress”) [13] [14]. These groups aimed to address any case of reported violence within 24 hours depending on the nature of the abuse and provided holistic services, including medical care, psycho-social/emotional counselling, nutrition support, shelter, legal support and documentation of events. Community committee members and community advisors were trained to advocate on violence redressal with the police and walk in to negotiate with the police for justice for victims of violence. Program lawyers were also contacted for cases requiring legal support and a booklet with details of lawyers who could be contacted in an emergency was distributed.

To build a supportive environment, the program advocated with multiple stakeholders who wield power, such as brothel owners, madams, pimps, brokers, lodge owners, auto drivers and FSWs’ regular sex partners, to create awareness of HIV prevention and the importance of condom use. Posters were displayed at police stations on sex worker-related issues, and videos were developed on safe sex and the need to protect sex workers from violence/abuse by clients and anti-social elements. Meetings were organized with district-level stakeholders such as the police, government officials and doctors from program clinics to advocate on issues affecting key populations, build rapport and seek support. In addition, a lawyers’ forum was established, and in each program district, two lawyers provided advice on key populations’ rights to help them protect themselves from violations.

## Methods

### Design, Setting and Sample

Data for this study were drawn from two rounds (2006 and 2009) of Behavior Surveillance Surveys (BSS), a cross-sectional study conducted among FSWs in six districts of Tamilnadu, India to monitor key components of the HIV prevention program. Inclusion criteria for participation in the survey were any women aged 18 years or older, who had engaged in sex work, either full-time or part-time, in the three months prior to the survey. Sample size was calculated to detect a 15% point increase in consistent condom use with clients from a baseline value of 50% at the district level with 80% power and alpha error of 5%. Overall, a sample of 2010 and 2500 FSWs in 2006 and 2009 respectively, completed the interview.

A multi-staged cluster sampling procedure was used for selection of participants in both rounds. The conventional cluster sampling approach was used for selecting FSWs based in non-public places (brothels, hotels, lodges, homes) and time-location cluster sampling method was used for selecting FSWs soliciting in public places (streets, market areas, highways and cinema halls). A list of hotspots (places where FSWs congregate to solicit clients) was developed through a rapid mapping exercise conducted using key informant interviews with community members and key local stakeholders such as the police and social workers. At each hotspot data were gathered on the presence of FSWs at different times of the day on different days of the week. This information was used to develop the list of primary sampling units for both conventional and time-location cluster sampling. Data collected during the mapping exercise were consolidated and finalized after discussion with the NGOs/community-based organizations (CBOs).

Hotspots located in areas covered by an NGO/CBO outreach worker for program implementation were grouped to form a stratum of 200 - 250 FSWs. Each stratum was further divided into two sampling frames of PSUs based on the typology of sex work (non-public place, public place). In each stratum, FSWs were recruited through a two-stage sampling procedure. In the first stage, a fixed number of PSUs were selected within each stratum using the proportion to population size procedure. The number of interviews to be conducted in each PSU was allocated proportionally. In the second stage, respondents were selected within each selected hotspot.

Face-to-face interviews were conducted by trained field investigators in the local language, Tamil, using a structured questionnaire that included questions on socio-demographic, sexual behavior, self-reported STI symptoms, treatment seeking behavior, and program exposure. Interviews were conducted in locations previously hired for data collection purposes.

### Ethical Considerations

Prior to administering the interview, written consent was obtained from all the participants after they were informed in detail about the survey and all their questions answered. No names or identifying information were collected.

### Measures

The outcome measures used in this study were: 1) consistent condom use with occasional clients, defined as condom use at every sexual encounter with occasional clients in the past year; 2) consistent condom use with regular clients, defined as condom use at every sexual encounter with regular clients in the past year; and 3) self-reported STI symptoms—participants were asked if they had experienced any of the following STI symptoms in the past 12 months: vaginal discharge, lower abdominal pain without diarrhea or during menstrual cycle, and genital ulcer or sores.

Exposure to the intervention was the key independent variable considered in the study, derived through a series of questions that were asked regarding contact by the program, membership in a CBO and service utilization. Those who were contacted by a peer educator or outreach worker at least three times in the past 6 months, had been taken by a peer educator to a program clinic for a health check-up in the past 6 months, had seen a demonstration of correct condom use, had visited a program supported clinic at least 2 - 3 times in the past 6 months, had attended a meeting or discussion at the program office in the past 6 months or were a registered CBO member were defined as exposed to the intervention.

The socio-demographic characteristics considered in this study included age (<30, 30 - 34, 35+ years), education status (illiterate, primary education, secondary education and above), current marital status (yes/no), engagement in occupations other than sex work (yes/no) and alcohol consumption in the past month (yes/no). The sex work characteristics considered in the study included duration in sex work (<4 years, 4 - 7 years, 8+ years) and primary place where clients are solicited (street, home, brothel-based). Both socio-demographic and sex work characteristics were used as covariates in the multivariate analyses.

### Statistical Analyses

Descriptive statistics were used to describe the population. Chi-square test was used to assess the difference between FSWs exposed to intervention and those who were not exposed in terms of their socio-demographic and sex work characteristics. A series of multivariate logistic regression models were constructed to examine the association between exposure to the intervention and change over time with condom use behavior and self-reported STI. All the analyses were performed using STATA 11.1.

## Results

Of the total sample, 48% in 2006 and 90% in 2009 reported exposure to intervention (not presented in tabular form). No significant difference was observed among the groups exposed and not exposed to the intervention in terms of age, education, and marital status in 2006. A higher proportion of FSWs exposed than those not exposed to the intervention reported engaging in occupations other than sex work and practicing sex work for a longer duration (4+ years) in 2006. Further, a significantly lower proportion of FSWs exposed to intervention reported alcohol consumption in the past month. In 2009 significant differences were observed between FSWs exposed and not exposed to the intervention in terms of education, marital status, engagement in occupations other than sex work, duration in sex work and past month alcohol consumption (Table 1).

**Table 1:** Socio-demographic profile and sex work characteristics by exposure to intervention among female sex workers, Tamil Nadu, India, 2006 and 2009.

Characteristics	2006 N = 2010			2009 N = 2500		
	Exposure to intervention			Exposure to intervention		
	No % (n)	Yes % (n)	P-value	No % (n)	Yes % (n)	P-value
**Age (years)**			0.188			0.307
<30	30.5 (322)	26.9 (257)		25.7 (66)	23.6 (529)	
30 - 34	30.6 (323)	33.1 (316)		28.4 (73)	33.1 (743)	
35+	38.9 (411)	39.9 (381)		45.9 (118)	43.3 (971)	
**Education status**			0.109			<0.001
Illiterate	30.1 (318)	26.5 (253)		24.9 (64)	17.2 (385)	
Primary education	25.3 (267)	24.5 (234)		13.6 (35)	21.9 (492)	
Secondary education +	44.6 (471)	49.0 (467)		61.5 (158)	60.9 (1366)	
**Currently married**	61.7 (651)	65.3 (623)	0.089	50.6 (130)	59.3 (1330)	0.007
**Engaged in occupations other than sex work**	53.9 (569)	58.3 (556)	0.047	74.7 (192)	55.7 (1249)	<0.001
**Duration in sex work (years)**			<0.001			0.014
<4	36.8 (389)	27.0 (258)		37.7 (97)	40.4 (906)	
4 - 7	40.4 (427)	49.6 (473)		42.0 (108)	46.1 (1034)	
8+	22.7 (240)	23.4 (223)		20.2 (52)	13.5 (303)	
**Typology of sex work**						0.504
Street-based	100.0	100.0	-	91.1 (234)	90.2 (2024)	
Home-based				7.0 (18)	6.5 (146)	
Brothel-based				2.0 (5)	3.3 (73)	
**Consumed alcohol past month**	85.1 (899)	77.2 (736)	<0.001	46.7 (120)	24.0 (538)	<0.001

* P-value based on Chi-square test of independence for categorical variables.

As seen in Table 2, in both the rounds FSWs who were exposed to the intervention were more likely to report consistent condom use with occasional (2006: adjusted OR = 1.90, 95% CI: 1.56 - 2.33; 2009: adjusted OR = 6.80, 95% CI: 5.0 - 9.24) and regular clients (2006: adjusted OR = 1.99, 95% CI: 1.62 - 2.44; 2009: adjusted OR = 9.51, 95% CI: 6.97 - 13.0) as compared to their respective counterparts. Compared to 2006, there was a considerable increase in the proportion of FSWs reporting consistent condom use with occasional and regular clients at follow-up. Further, the increase in consistent condom use over time with occasional clients (adjusted OR = 3.53, 95% CI: 2.47 - 5.05) and regular clients (adjusted OR = 4.97, 95% CI: 3.43 - 7.16) was higher among FSWs who were exposed to the intervention as compared to those who reported no exposure. Additionally, a significant decline was observed in self-reported STI overtime, among FSWs who reported exposure to intervention compared to their counterparts (adjusted OR = 0.39, 95% CI: 0.26 - 0.59).

**Table 2:** Effect of intervention exposure on condom use and self-reported sexually transmitted infection symptoms overtime among female sex workers, Tamil Nadu, India, 2006-2009.

	2006		2009			
Outcome measures	Exposure to intervention		Exposure to intervention		Time x exposure adjusted OR (95% CI)	P-value
	No	Yes	No	Yes		
**Condom use with occasional clients—% (N)**	62.9 (1051)	76.4 (948)	44.4 (252)	86.0 (2210)		
**Condom use with occasional clients—AOR (95% CI)**	Ref	**1.90 (1.56, 2.33)**	Ref	**6.80 (5.0, 9.24)**	**3.53 (2.47, 5.05)**	<0.001
**Condom use with regular clients—% (N)**	63.4 (981)	77.7 (945)	39.7 (242)	88.8 (2005)		
**Condom use with regular clients—AOR (95% CI)**	Ref	**1.99 (1.62, 2.44)**	Ref	**9.51 (6.97, 13.0)**	**4.95 (3.43, 7.16)**	<0.001
**Self-reported STI symptoms—% (N)**	17.3 (1056)	17.3 (954)	22.2 (257)	8.6 (2243)		
**Self-reported STI symptoms—AOR (95% CI)**	Ref	1.0 (0.79, 1.27)	Ref	**0.42 (0.29, 0.60)**	**0.39 (0.26, 0.59)**	<0.001

OR: odds ratio; CI: confidence interval.

## Discussion

This study presents findings on the effect of an up scaled HIV prevention program for FSWs in the state of Tamilnadu, India. The results indicate a strong association between exposure to the intervention and a reduction in HIV risk with regard to condom use and self-reported STI. Our findings show that FSWs exposed to the intervention were significantly more likely to report consistent condom use with both regular and occasional clients. Further, a significant decline in self-reported STI was observed among FSWs reporting program exposure. These findings are in line with results from assessments of HIV prevention programs with FSWs in other high HIV prevalence states of India, indicating improvements in condom use with commercial partners and a decline in STI [7] [15] [16] [17].

As highlighted in this study, there was a dramatic increase in program exposure between 2006 and 2009. Possible reasons for increased exposure could be the rapid scale-up of the Avahan program in Tamilnadu and the involvement of the community in all aspects of program implementation. While initially experts from program implementing NGOs conducted program monitoring, under Avahan, the approach was to engage community members in monitoring activities with supportive supervision from NGO staff. As reported in other assessments of Avahan [18] [19], peer outreach workers were compensated for their time in the project, made responsible for outreach service delivery and management, provided microplanning tools and given well-defined targets, which resulted in a marked increase in the number of key populations contacted by the program, narrowed the condom distribution gap among key populations and increased clinic utilization.

The increase in consistent condom use may be the result of several factors. Along with peer educators motivating FSWs to adopt safer sex behaviors through regular risk reduction counselling, condom use demonstrations and building FSWs’ negotiation skills, the program was also able to meet the minimum requirements for condoms through the distribution of condoms by peer educators, as well as social marketing to cover the estimated number of commercial sex acts. Improving access to condoms when needed may have contributed to the reported improvement in condom use with commercial partners over the stu1dy period, as has been observed in other states which adopted a similar intervention approach [16] [20]. Additionally, the mobilization of community members and setting up crisis response groups may have provided FSWs’ the structural support to address violence from clients, and insist on condom use [21].

Due to the lack of information on clinically-diagnosed STIs, self-reported STIs were taken as a proxy indicator. Similar to findings from assessments of interventions with FSWs in other high prevalence states of India, a significant decline in self-reported STI symptoms was observed in the current study [16] [20]. Prevention of STI transmission to and from sex workers is critical for HIV prevention; and as mentioned earlier, one of the core components of the TAI-VHS program was the scaling up of HIV prevention services with a focus on STI treatment. FSWs were encouraged to visit program clinics for health and STI check-ups at least one in three months. Regular screening and treatment of symptomatic and asymptomatic STIs has proven effective in reducing STI rates in prior research [22], and could have led to the decline in self-reported STI overtime in this state as well. In addition to providing a range of preventive services, DIC also provided other services, such as a food bank and cloth bank for those in need, and entertainment activities, which may have resulted in a large number of sex workers spending time at the DIC, and consequently an increased clinic flow. Further, peer outreach workers were trained to counsel FSWs on the importance of STI testing and treatment; at the same time, FSWs were motivated to screen for STI by peer outreach workers and community volunteers during routine outreach activities, and healthcare providers during clinic visits, which may have also resulted in an increased uptake of STI testing and treatment services [23]. Frequent testimonial sharing by FSWs regarding clinic visits and experiences during group counselling sessions conducted by peer educators may have also motivated sex workers to come forward for health check-ups and STI screening. Setting up of “safe” spaces for communities at government-run clinics staffed by community members, and efforts to build acceptance of key populations at the facility level through the display of posters and by screening videos may have helped to reduce stigma at and improve STI screening. Community mobilization efforts may have also built self-efficacy for service utilization [24] [25].

The significant reduction in self-reported STI and high exposure to the intervention also indicate that the HIV prevention program is reaching FSWs, and safer behaviors are being adopted, as observed in the current study where a higher proportion of FSWs exposed to the intervention as compared those with no exposure reported using condoms.

The need to build a supportive environment to address the distal factors of HIV vulnerability, including stigma and violence for effective HIV prevention is well documented [26] [27]. In Tamilnadu, the program made concerted efforts through advocacy with multiple stakeholders, including the police, government officials, local lawyers, brothel owners, pimps, and FSWs’ regular sex partners, to create an enabling environment for key populations, who are at a high risk for acquiring and transmitting HIV, to adopt safer behaviors [6]. As seen in other studies from India, efforts to empower FSWs to address structural barriers and form community groups through community mobilization can result in substantially lower STI risk and higher likelihood of seeking STI treatment from health facilities as compared to those not collectivized [25] [28]; setting up crisis response groups to redress violence perpetuated by partners and others could have also helped to build social capital and a supportive network among FSWs to negotiate condom use with all clients and reduce their HIV risk [29].

While this study highlights a strong association between exposure to the TAI-VHS HIV prevention intervention and safer sex behaviors and lower self-reported STI, the study results should be interpreted in light of certain limitations. First, the 2006 survey was conducted when the HIV prevention program was already underway, and hence it was not a true baseline. Second, the dependent variables considered in this paper were based on self-reported responses, which may be biased due to socially desirable responses from participants. However, the use of experienced researchers may have reduced this bias to some extent. Finally, the study findings cannot be generalized to all FSWs across India as sex work in India is characterized by inter- and intra-regional differences. However these limitations do not compromise the internal validity of the data. Our study findings are consistent with results from other assessments indicating an improvement in condom use behavior with commercial clients and reduction in STI following upscaled HIV prevention programs [17] [19] [30] [31].

In short, our study shows that the HIV prevention program in Tamilnadu resulted in improvements in consistent condom use with regular and occasional clients, and a decrease in self-reported STI among FSWs exposed to intervention. These findings suggest that HIV prevention programs must aim to saturate coverage among key populations to sustain the gains achieved in reducing HIV risk.

## Notes

**Submit or recommend next manuscript to SCIRP and we will provide best service for you:**

Accepting pre-submission inquiries through Email, Facebook, LinkedIn, Twitter, etc.

A wide selection of journals (inclusive of 9 subjects, more than 200 journals)

Providing 24-hour high-quality service

User-friendly online submission system

Fair and swift peer-review system

Efficient typesetting and proofreading procedure

Display of the result of downloads and visits, as well as the number of cited articles

Maximum dissemination of your research work

Submit your manuscript at: http://papersubmission.scirp.org/ Or contact wja@scirp.org
